# Supplementary material for: Bamboo tea: reduction of taxonomic complexity and application of DNA diagnostics based on rbcL and matK sequence data
Source: PeerJ. 2016 Dec 8;4:e2781. doi: 10.7717/peerj.2781 (PMC5149056; doi:10.7717/peerj.2781)
Supplement: Supplemental Information 1 [file peerj-04-2781-s001.pdf]

SUPPLEMENTARY TABLES

LIST OF TABLES

1 List of universal and diagnostic primer 1
3 Genbank sequence accessions (cytoplasmic) 1
4 Genbank sequence accessions (nuclear) 2
2 List of secondary specimens (lemongrass and carnation) 5
5 Anatomical diagnostic key 6
6 BLOG bamboo tribe classification results 7

Table 1. List of universal and diagnostic (ARMS) primer used in the current study. The table includes primer names and sequences, melting temperatures (Tm) of each individual primer, the annealing temperatures (An) for each primer pair and references to corresponding publications.

Name	Sequence (5'...3')	Tm (°C)	An (°C)	Design
rbcLa_f[u]	ATGTCACCACAAACAGAGACTAAAGC	62.2	58	Kondo et al. [1996]
rbcLa_rev[u]	GTAAAATCAAGTCCACCRCG	74.7		Fofana et al. [1997]
rbcLb-Sf[u]	AGACCTTTTTGAAGAAGGTTCTGT	62.2	55	Dong et al. [2014]
rbcLb-Sr[u]	TCGGTCAGAGCAGGCATATGCCA	74.7		
1R_KIM_r[u]	ACCCAGTCCATCTGGAAATCTTGGTTC	72.1	52	Ki-Joong Kim[p]
3F_KIM_f[u]	CGTACAGTACTTTTGTGTTTACGAG	60.6		
ITS5[u]	GGAAGTAAAAGTCGTAACAAGG	58.4	56	White et al. [1990]
ITS4[u]	TCCTCCGCTTATTGATATGC	61.5		
Bbo-C-407-r[d]	AGGBGGRCCTSGRAAAGTTTT<u>C</u>GA	58.7		current study
Dian-T-223-r[d]	CGGGCTCGATGTGGTAG<u>T</u>AA	64.9		
Cym-A-254-r[d]	AGCTACATAACAGATATATTGATC<u>A</u>GG	59.5		

[d] diagnostic primer (ARMS), destabilizing nucleotide underlined
[u] universal primer
[p] unpublished

Table 3. GenBank sequence accessions of two plastid DNA regions (rbcL and matK) used in the current study.

Taxon	rbcL	matK
Arundinarieae		
Arundinaria gigantea subsp. tecta	AJ746179	EF125165
Borinda emeryi	EF125079	EF125167
Chimonobambusa marmorea	AJ746176	EF125168
Drepanostachyum falcatum	AJ746265	EF125170
Fargesia dracocephala	AJ746266	KP685610
Fargesia sp. Asmussen	AM110249	AM114722
Indocalamus latifolius	AJ746177	EF125173
Phyllostachys aurea	HE573324	AF164390
Phyllostachys bambusoides	AB088833	AB088805
Phyllostachys nigra	HE573325	EU434241
Pleioblastus maculatus	JN247242	JN247148
Pseudosasa amabilis	AJ746273	KP093752
Pseudosasa japonica	FN870405	HG794001
Thamnocalamus spathiflorus	EF125087	KP685639
Bambubseae		
Bambusa multiplex	M91626	EF125166

Table 3. GenBank sequence accessions of two plastid DNA regions (rbcL and matK) used in the current study. (continued)

Taxon	rbcL	matK
Bambusa valida	AJ746171	EU434255
Bambusa vulgaris	HE573326	JX428392
Chusquea culeou	HE575810	HE575860
Greslania multiflora	HE575811	HE575861
Melocanna baccifera	EF125082	EF125174
Olmeca recta	AJ746269	HQ847275
Otatea acuminata	AJ746271	HQ847256
Thyrsostachys siamensis	EF125088	EU434261
Olyreae		
Cryptochloa strictiflora	JN205319	EU434281
Olyra latifolia	EF125090	AF164386
Pariana radiciflora	AY632369	AF164387
Oryzoideae		
Oryza sativa Japonica Group	KF731173	KF731059
Porteresia coarctata	HE577876	AF148669
Panicoideae		
Cymbopogon citratus	EF125117	GQ434093
Cymbopogon goeringii	KC164298	KF163712
Lophatherum gracile	HM167472	JN407183
Pooideae		
Triticum aestivum	AY328025	AF164405
Triticum dicoccon	LN626623	HQ894423
Triticum monococcum	AY836162	HM540031
Caryophyllaceae		
Silene baccifera	KM360741	JF956236
Silene conica	EF418564	FJ589520
Silene gallica	HM850354	FJ589528
Silene otites	KC171366	FJ589547
Silene pendula	EF418561	FJ589550
Silene schafta	EF418563	FJ589556
Silene vulgaris	KT178142	EU749399

Table 4. GenBank sequence accessions of one nuclear DNA region (ITS) used in the current study.

Taxon	ITS
Caryophyllaceae	
Dianthus albens	GU440775
Dianthus algetanus	GU440776
Dianthus anatolicus	GU440777
Dianthus andrzejowskianus	JN589032
Dianthus anticarius	GU440778
Dianthus armeria	GU440780
Dianthus barbatus	GU440783
Dianthus benearnensis	GU440784
Dianthus biflorus	GU440785

Table 4. GenBank sequence accessions of one nuclear DNA region (ITS) used in the current study. (continued)

Taxon	ITS
Dianthus bolusii	GU440786
Dianthus broteri	GU065884
Dianthus broteroi	JN589117
Dianthus caespitosus	GU440789
Dianthus capitatus	GU440792
Dianthus carthusianorum	GU440794
Dianthus caryophyllus	JN589053
Dianthus caucaseus	JN589024
Dianthus charidemi	GU440795
Dianthus chinensis	GU440796
Dianthus corymbosus	GU440801
Dianthus costae	GU440802
Dianthus cretaceus	GU440804
Dianthus crinitus	GU440806
Dianthus cruentus	GU440807
Dianthus cyri	GU440808
Dianthus deltoides	GU440809
Dianthus diffusus	GU440811
Dianthus eretmopetalus	GU440813
Dianthus excelsus	GU440815
Dianthus ferrugineus	GU440803
Dianthus giganteiformis	GU440818
Dianthus giganteus	GU440819
Dianthus glacialis	GU440821
Dianthus gracilis	GU440822
Dianthus gratianopolitanus	JQ307857
Dianthus haematocalyx	GU440824
Dianthus hyssopifolius	GU440826
Dianthus imereticus	JN589026
Dianthus inamoenus	JN589070
Dianthus integer	GU440827
Dianthus knappii	GU440828
Dianthus laingsburgensis	GU440829
Dianthus langeanus	JQ307858
Dianthus libanotis	GU440835
Dianthus lusitanus	GU440838
Dianthus micranthus	GU440839
Dianthus microlepis	GU440840
Dianthus micropetalus	GU440841
Dianthus mooiensis	GU440842
Dianthus multiceps subsp. multiaffinis	GU440843
Dianthus myrtinervius	GU440845
Dianthus namaensis	GU440846
Dianthus orientalis	GU440847
Dianthus pungens	GU440852
Dianthus pygmaeus	GU440853
Dianthus repens	JN589124
Dianthus seguieri	JN589106
Dianthus serotinus	GU440857
Dianthus serratifolius	GU440858
Dianthus serrulatus	GU440859

Table 4. GenBank sequence accessions of one nuclear DNA region (ITS) used in the current study. (continued)

Taxon	ITS
Dianthus sinaicus	JQ307884
Dianthus sp. cdK73	FJ593175
Dianthus squarrosus	JN589065
Dianthus stenopetalus	GU440861
Dianthus strictus	GU440862
Dianthus strymonis	GU440863
Dianthus superbus	FJ980409
Dianthus sylvestris	GU440870
Dianthus tenuiflorus	GU440871
Dianthus trifasciculatus	GU440874
Dianthus tripunctatus	GU440875
Dianthus viscidus	GU440879
Dianthus webbianus	GU440814
Dianthus zeyheri	GU440881
Dianthus zonatus	GU440882
Ixoca quadrifida	JX274534
Lychnis coronata	JX274529
Melandrium keiskei	DQ908643
Silene dianthoides	KJ918494
Silene jenisseensis	KF267895
Silene multicaulis	KF267891
Silene multiflora	KF274511
Silene occidentalis	DQ908656
Silene sibirica	JX274521
Silene uralensis subsp. apetala	JX274519
Silene vulgaris	KJ918500

REFERENCES

Dong, W., Cheng, T., Li, C., Xu, C., Long, P., Chen, C., and Zhou, S. (2014). Discriminating plants using the DNA barcode rbcLb: An appraisal based on a large data set. *Molecular Ecology Resources*, 14(2):336–343.

Fofana, B., Harvengt, L., Baudoin, J. P., and Jardin, P. D. U. (1997). New Primers For The Polymerase Chain Amplification Of cpDNA Intergenic Spacers In Phaseolus Phylogeny. *Belgian Journal of Botany*, 129(1997):118–122.

Kondo, K., Terabayashi, S., Okada, M., Yuan, C., and He, S. (1996). Phylogenetic relationship of medicinally important Cnidium officinale and Japanese Apiaceae based on rbcL sequences. *Journal of Plant Research*, 109(1982):21–27.

White, T. J., Bruns, S., Lee, S., and Taylor, J. (1990). Amplification and direct sequencing of fungal ribosomal RNA genes for phylogenetics. In *PCR Protocols: A Guide to Methods and Applications*, pages 315–322. Academic Press, Inc.

Table 2. Specimen accessions (Acc) of lemongrass (*C.*, *Cymbopogon*, *Panicoideae*, L1 - L2) and carnation (*D.*, *Dianthus*, *Caryophyllaceae*, D1 - D10) their taxon names and GenBank sequence accessions of three plastid DNA regions (rbcLa, rbcLb and matK-KIM) and for carnation accessions also for one nuclear DNA region (ITS).

Acc	Taxon	rbcLa	rbcLb	matK-KIM	ITS
	Panicoideae				
L1	*C. citratus*	KU722908	KU748525	KU722906	
L2	*C. citratus*	KU722907	KU748524	KU722905	
	Caryophyllaceae				
D1	*D. caryophyllus*	KU722895	KU722853	KU722867	KU722881
D2	*D. chinensis*	KU722896	KU722854	KU722868	KU722882
D3	*D. deltoides*	KU722898	KU722856	KU722870	KU722884
D4	*D. deltoides*	KU722897	KU722855	KU722869	KU722883
D5	*D. gratianopolitanus*	KU722899	KU722857	KU722871	KU722885
D6	*D. imereticus*	KU722900	KU722858	KU722872	KU722886
D7	*D. longicalyx*	KU722901	KU722859	KU722873	KU722887
D8	*D. superbus*	KU722902	KU722860	KU722874	KU722888
D9	*D. superbus*	KU722903	KU722861	KU722875	KU722889
D10	*D. turkestanicus*	KU722904	KU722862	KU722876	KU722890

Table 5. Anatomical diagnostic key used to microscopically differentiate between Bamboo, lemongrass and Carnation leaf fragments present in Bamboo tea products. General features (GF) of adaxial and abaxial epidermis cells and cell walls (CW) are described in addition to characteristics of bulliforme cells (BC), stomata (ST), trichomes (TR) and wax structures (WS). Further features are derived from trichomes of the leaf margin (TM) and structures of the mesophyll (MP).

		Bamboo	Lemongrass	Carnation
Epidermis-ad	GF	long cells, sinuous curved CW and short cells alternately		polygonal cells, mostly straight CW
	BC	10-30 x 10-20 um CW polygonal, thick, straight to slightly sinuous curved	70 x 30 um CW polygonal, thick, straight	none
	ST	none or dumbbell-shaped guard cells	dumbbell-shaped guard cells	diacytic / anisocytic , numerous
	TR	none or unicellular, short, thick-walled, acuminate	unicellular, short, thick walled, acuminate 1-2 celled, short, minute, thin-walled, papillate	none or a few, unicellular, short, thick walled
	WS	inconspicuous		
Epidermis-ab	GF	long cells, sinuous curved CW and short cells alternately		polygonal cells, mostly straight CW
	BC	none	none	none
	ST		more than adaxial	
		dumbbell-shaped guard cells difficult to see because of wax papilla	dumbbell-shaped guard cells conspicuous	diacytic / anisocytic
	TR	unicellular, long / short, thick-walled, acuminate 1-2 celled, short, minute, thin-walled, papillate	unicellular, short, thick-walled, acuminate	none or unicellular, thick-walled
	WS	papilla	inconspicuous	
TM		mostly unicellular, short, thick-walled, acuminate		1-3 celled, short acuminate mostly unicellular, short, acuminate
MP		main veins running parallel		reticulate
		vein pattern tessellate, visible at 40x magnification [a]	only main veins visible at 40 x magnification glandular cells	many relatively large crystal druses along main veins and in intercostal regions

[a] except *Bambusa*

Table 6. BLOG bamboo tribe (Aru = *Arundinarieae*, Bam = *Bambuseae*, Oly = *Olyreae*) classification results. BLOG results using bamboo tribes for classification with single marker datasets (*rbcLa*, *rbcLb*, *matKK*) and combined marker datasets (*rbcL*: *rbcLa* + *rbcLb*, *rbcL+matK*: *rbcL* + *matKK*). The diagnostic (LOGic) formulas cover (C) a certain proportion of the sequences, ideally 100 %. Proportion of false positive (FP) and negative (FN) classifications as well as the ratio false to true positive (TP) classifications are shown. The score refers to the Laplace score

Classes	C (%)	FN (%)	FP (%)	Score	FP/TP	Formulas
			**rbcLa**			
Aru	96.0	4.0	0.0	0.93	0.00	pos45=T
Bam	100.0	0.0	3.6	0.80	0.09	pos234=C AND pos538=T
Oly	100.0	0.0	0.0	0.67	0.00	pos526=T
			**rbcLb**			
Aru	100.0	0.0	0.0	0.93	0.00	pos343!=G AND pos497!=G
Bam	100.0	0.0	0.0	0.86	0.00	pos204=C AND pos771=C
Oly	100.0	0.0	0.0	0.67	0.00	pos546=A
			**rbcL**			
Aru	100.0	0.0	0.0	0.93	0.00	pos252=G AND pos1103!=C
Bam	100.0	0.0	0.0	0.86	0.00	pos526=C AND pos1103=C
Oly	100.0	0.0	0.0	0.67	0.00	pos878=A
			**matKK**			
Aru	100.0	0.0	0.0	0.93	0.00	pos632=G
Bam	100.0	0.0	0.0	0.86	0.00	pos667=G AND pos688=A
Oly	100.0	0.0	0.0	0.67	0.00	pos536=A
			**rbcL + matK**			
Aru	100.0	0.0	0.0	0.93	0.00	pos1788=G
Bam	100.0	0.0	0.0	0.86	0.00	pos1485=C AND pos1844=A
Oly	100.0	0.0	0.0	0.67	0.00	pos1692=A
