# Supplementary material for: Bamboo tea: reduction of taxonomic complexity and application of DNA diagnostics based on rbcL and matK sequence data
Source: PeerJ. 2016 Dec 8;4:e2781. doi: 10.7717/peerj.2781 (PMC5149056; doi:10.7717/peerj.2781)

SUPPLEMENTARY FIGURES

LIST OF FIGURES

1 Barcoding with LOGic formulas (BLOG) analysis of single and combined bambusoid DNA marker data in comparison. The proportion of coverage (C) and false negatives (FN) using logic formulas is shown as barplot (blue/red). The Laplace score (Score) is indicated by a dashed line and in case there are false positives (FP) detected evaluating a dataset they are indicated by red triangles. 2

Figure 1. Barcoding with LOGic formulas (BLOG) analysis of single and combined bambusoid DNA marker data in comparison. The proportion of coverage (C) and false negatives (FN) using logic formulas is shown as barplot (blue/red). The Laplace score (Score) is indicated by a dashed line and in case there are false positives (FP) detected evaluating a dataset they are indicated by red triangles.

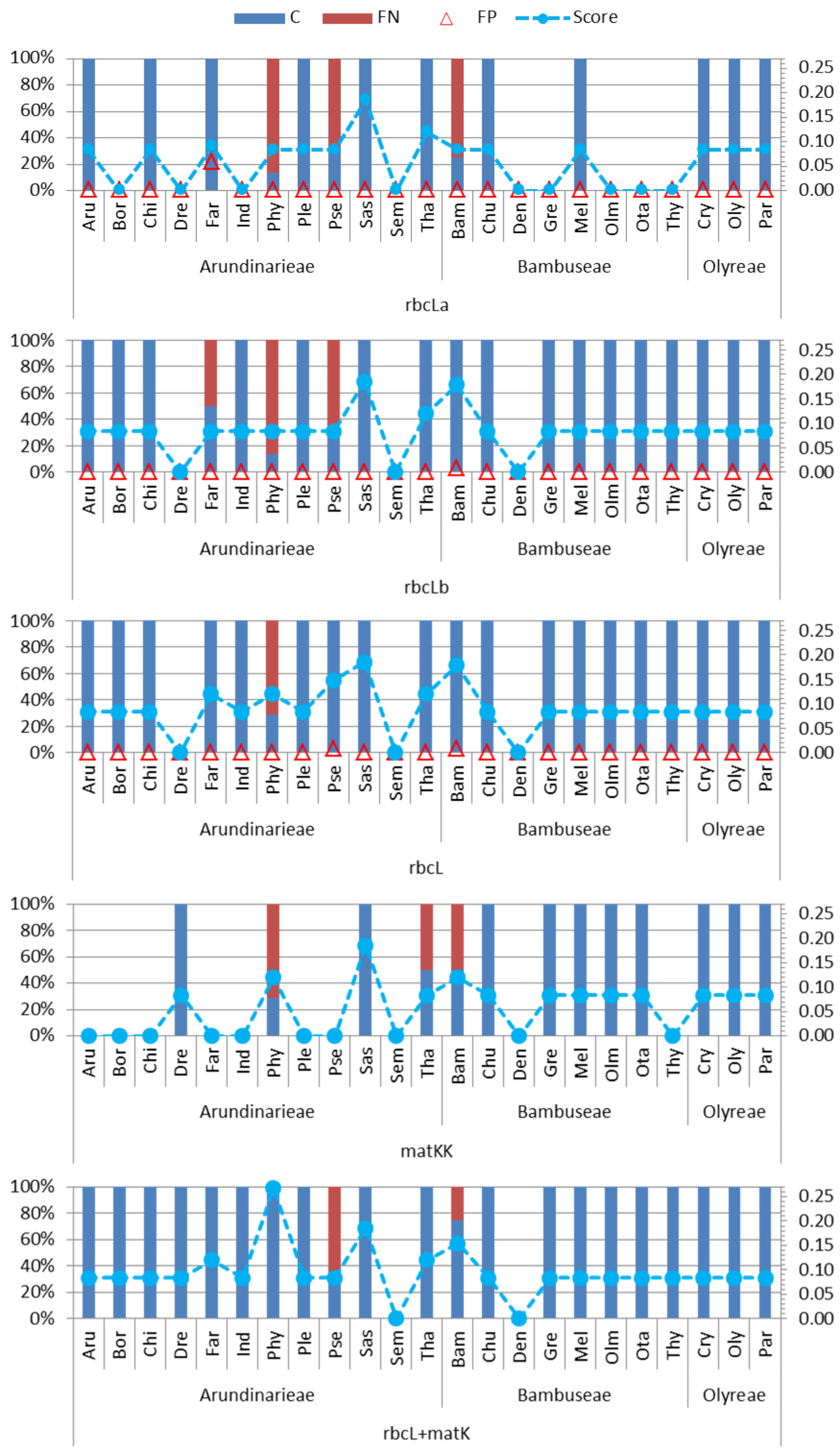

Supplement: Supplemental Information 2 [file peerj-04-2781-s002.pdf]
